# Adherence to recommendations for ART and targeted PrEP use among HIV serodiscordant couples in East Africa: the “PrEP as a bridge to ART” strategy

**DOI:** 10.1186/s12889-020-09712-3

**Published:** 2020-10-28

**Authors:** Nicholas Musinguzi, Lara Kidoguchi, Nelly R. Mugo, Kenneth Ngure, Elly Katabira, Connie L. Celum, Jared M. Baeten, Renee Heffron, Jessica E. Haberer, on behalf of the Partners Demonstration Project Team

**Affiliations:** 1 Global Health Collaborative, Mbarara University of Science and Technology, P. O Box 434, Mbarara, Uganda; 2 Department of Medicine, University of Washington, Seattle, USA; 3 Centres for Clinical Research, Kenya Medical Research Institute, Nairobi, Kenya; 4 Department of Global Health, University of Washington, Seattle, USA; 5 School of Public Health, Jomo Kenyatta University of Agriculture and Technology Nairobi, Nairobi, Kenya; 6 Infectious Disease Institute, Makerere University, Kampala, Uganda; 7 Department of Epidemiology, University of Washington, Seattle, USA; 8 Massachusetts General Hospital and Harvard Medical School, Boston, USA

**Keywords:** PrEP, ART, Adherence, Demonstration project, Kenya, Uganda

## Abstract

**Background:**

PrEP use should be aligned with periods of risk for HIV acquisition. For HIV serodiscordant couples, PrEP can be used as a bridge until the partner living with HIV takes antiretroviral therapy (ART) long enough to achieve viral suppression (the “PrEP as a Bridge to ART” strategy). However, adherence to this strategy is unknown.

**Methods:**

In a demonstration project in Kenya and Uganda, HIV-uninfected partners of serodiscordant couples were advised to take PrEP until the partner living with HIV took ART for ≥ 6 months. PrEP discontinuation was then recommended unless there were concerns about ART adherence, immediate fertility intentions, or outside partners with unknown HIV/ART status. Electronic adherence monitoring and socio-behavioral questionnaire data were used in logistic regression models to explore completion of this strategy and continuation of PrEP beyond recommendations to stop its use.

**Results:**

Among 833 serodiscordant couples, 436 (52%) HIV-uninfected partners completed ≥ 6 months of PrEP as a bridge to ART. Strategy completion was associated with older age (aOR per 5 years = 1.1; *p* = 0.008) and having fewer children (aOR = 0.9; *p* = 0.019). Of the 230 participants encouraged to stop PrEP according to strategy recommendations, 170 (74%) did so. PrEP continuation among the remaining 60 participants was associated with more education (aOR = 1.1; *p* = 0.029), a preference for PrEP over ART (aOR = 3.6; *p* = 0.026), comfort with managing their serodiscordant relationship (aOR = 0.6; *p* = 0.046), and believing PrEP makes sex safe (aOR = 0.5; p = 0.026).

**Conclusion:**

Half of participants completed the PrEP as a bridge to ART strategy and the majority stopped PrEP as recommended. These findings suggest that targeting PrEP to periods of risk is a promising approach; however, tailoring counseling around aligning PrEP use and HIV risk will be important for optimal strategy implementation.

## Background

Oral pre-exposure prophylaxis (PrEP) is efficacious in preventing HIV transmission in the context of sufficient adherence [1–3]. Unlike antiretroviral therapy (ART) for the treatment of HIV infection, PrEP use does not need to be lifelong. Rather, it can be aligned to periods of risk for HIV acquisition [4, 5]. That is, PrEP may be taken when an individual is at risk of infection and stopped when he or she is no longer at risk. This concept, known as prevention-effective adherence [6], may help avoid unnecessary costs and other burdens associated with daily pill taking for the individual and for the healthcare system.

Prevention-effective adherence, however, is not without its challenges in implementation. For example, the period of HIV risk may be uncertain. Although HIV serodiscordant couples face the potential for HIV transmission, the risk may be negligible if the partner living with HIV has a suppressed viral load (i.e., undetectable equals untransmittable, or U=U) [7]. Yet in practice, in many resource-limited settings, viral load monitoring is not readily available and may reduce confidence in treatment as prevention [8, 9]. Likewise, the risk of HIV infection may be present even in the setting of U=U, if a sexual partner outside the couple has HIV [10, 11]. Confidence in recommendations to stop PrEP may therefore be lacking.

Numerous other factors may also affect adherence to PrEP independent of the recommended timing. For instance, prior research in the Partners Demonstration Project, which involved serodiscordant couples in Kenya and Uganda, indicated that effective adherence to PrEP (defined as 6 or more doses per week) can be influenced by the frequency of sexual activity, comfort with daily PrEP, fertility intentions, age, desire for relationship success, relationship stability, and problematic alcohol use [5]. Strategies to align PrEP use with risk must therefore consider the perceptions, beliefs and behaviors contributing to both HIV risk and medication adherence.

The Partners Demonstration Project involved a public health-oriented PrEP delivery strategy consistent with prevention-effective adherence. Specifically, PrEP was recommended for the uninfected partner until the partner living with HIV had used ART for six months, a time period consistent with U=U [12]. The uninfected partner was then encouraged to discontinue PrEP use if he or she had no concerns about the partner’s ART adherence, had no immediate fertility intentions, or reported no other partners with unknown HIV/ART status. This PrEP delivery strategy has been called “PrEP as a Bridge to ART”.

In this paper, we investigated adherence to PrEP recommendations according to the PrEP as a Bridge to ART strategy. Our objectives were to 1) determine which participants completed 6 months of PrEP use; 2) determine which participants continued PrEP after receiving recommendations to stop its use; and 3) assess factors associated with each of these two populations. These data may help public health specialists in implementation of PrEP strategies consistent with prevention-effective adherence.

## Methods

### The partners demonstration project

The Partners Demonstration Project was a prospective, open-label study of ART and PrEP for HIV prevention among high risk mutually disclosed, HIV serodiscordant couples in Kenya and Uganda that took place between November 2012 and June 2016. Study procedures have been described elsewhere [12]. Briefly, HIV serodiscordant couples were recruited through referrals from voluntary counseling and testing centers, antenatal clinics, ART clinics, and through community outreach events that promoted couples-based HIV testing. Couples were eligible for enrolment if they were ≥ 18 years of age, sexually active, and intending to remain as a couple for ≥ 1 year. Because the goal of the study was to recruit couples at high risk of HIV infection and therefore more likely to benefit from the intervention, couples were eligible if they scored at least 5 points on a validated risk scoring tool [13, 14]. At enrollment, partners living with HIV had WHO stage I or II and were not yet using ART. Study visits were scheduled at 1 month, 2 months, and every 3 months after enrolment for up to 2 years. At these visits, participants were tested for HIV, PrEP was dispensed, and socio-behavioral questionnaires were administered to the couple. The above-noted PrEP as a Bridge to ART strategy was implemented by counselors highly experienced in working with serodiscordant couples. As described elsewhere [15], the counselors developed and refined key messages mapping to common participant concerns about the delivery of integrated PrEP and ART for HIV prevention. Of note, viral loads were not available during the conduct of the study, as is consistent with most provision of ART in resource-limited settings [8, 9] and did not influence decisions regarding the timing of PrEP use. The study estimated a 95% reduction in HIV transmission with only four incident seroconversions [12].

### Adherence measurement

PrEP adherence was monitored electronically using the medication event monitoring system (MEMS, WestRock, Switzerland), a pill bottle cap that records the date and time of opening. Adherence was computed as the total number of openings divided by the total number of expected openings during the days for which PrEP had been dispensed and when the participant was not on a protocol- related drug hold (e.g., for a side effect). Openings by study staff were censored from the adherence computation. Participants were considered lost to follow-up if they missed two consecutive study visits (i.e., about 6 months of no study contact).

### Study outcomes

This analysis assessed two outcomes:

1. Completion of the 6-month PrEP as a Bridge to ART strategy. Participants were considered to have completed the PrEP as a Bridge to ART strategy if their electronic adherence data indicated continuous PrEP use (i.e., no breaks of > 28 days) for ≥ 6 months following the date the partner living with HIV started ART or until the couple reported no longer being together due to a breakup or death (whichever occurred first). We considered > 28-day breaks in adherence, which are consistent with the 28-day interval for pharmacy refills. Shorter thresholds could be considered but are more likely to reflect poor adherence rather than discontinuation of PrEP. Participants who were lost to follow-up prior to the anticipated completion date were considered to have not completed the strategy. Additionally, couples were excluded if the partner living with HIV started ART < 6 months prior to the end of study follow-up.
2. Continuation of PrEP beyond recommendations to stop. Participants were considered to have continued PrEP use beyond recommendations to stop if they were encouraged to stop but self-reported continuation or if their electronic adherence data indicated PrEP use ≥ 9 months after the partner living with HIV had started ART. Participants are excluded from this outcome if they were not encouraged to stop PrEP due to reported concerns about the partner’s ART adherence, immediate fertility intentions, or other partners with unknown HIV/ART status. The 9-month threshold was chosen because study visits may not have coincided precisely with ART initiation. Note that this outcome only pertains to those participants who completed the PrEP as a Bridge to ART strategy per the above definition. Participants who were lost to follow-up after completion of the PrEP as a Bridge to ART strategy and had < 9 months of electronic adherence data following their partner initiating ART were considered to have stopped PrEP according to recommendations (N.B., PrEP was generally not otherwise available during the study period).

### Statistical analysis

From the prior research on PrEP adherence within the Partners Demonstration Project and other review of the literature, the following factors were considered as potentially associated with PrEP use and were therefore considered in the univariable models: gender [5], age [4, 16, 17], fertility intentions [5], problematic alcohol use [5, 16], depression [18], relationship status and satisfaction [5, 19], polygamous relationship [16], attitudes towards PrEP [5], concerns about ART adherence and viral suppression [5, 12], sex within the couple with < 100% condom use [5], use of other medications [20], number of biological children between the couple [17], and awareness of discordance status at study enrolment [19]. To assess factors associated with completion of the PrEP as a Bridge to ART strategy and those associated with continuation of PrEP beyond recommendations to stop, we used logistic regression with robust standard errors. All factors with a univariable *p* value < 0.10 were entered into the multivariable model. Only baseline covariates were considered.

### Ethics statement

The Division of Human Subjects at the University of Washington and the ethics review committees at each study site reviewed and approved the study protocol. All participants provided written informed consent.

## Results

### Participant characteristics

**Fig. 1:**
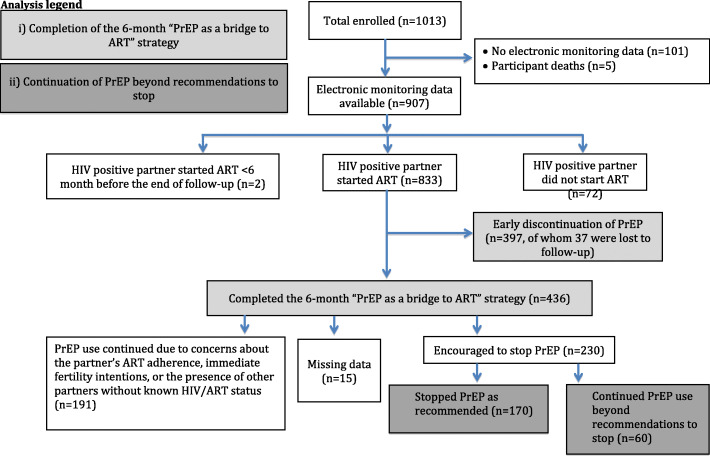
Evaluation of PrEP use per the PrEP as a Bridge to ART strategy. The light gray boxes indicate the populations compared in the first analysis, while the dark gray boxes indicate the populations compared in the second analysis

As shown in Fig. 1, 1013 couples were enrolled in the Partners Demonstration Project. Five participants died and 101 did not have electronic adherence monitoring data. Of the remaining 907 couples, 72 of the partners living with HIV did not initiate ART and two initiated ART < 6 months before the end of study follow-up, leaving 833 couples eligible for this analysis. Thirty-seven participants were lost to follow-up prior to completing the PrEP as a Bridge to ART strategy.

Just over one-third of analyzed participants taking PrEP were female (36%), the median age at screening was 30 years (IQR 26, 36), and the median education level was 8 years (IQR 6, 12), as shown in Table 1. Most (*n* = 675, 82%) had known their discordance status for > 1 month before enrollment and 665 (80%) preferred to take daily PrEP as opposed to having their partner take ART. Fertility intention was high (74%).

**Table 1:** Enrolment characteristics

Characteristics	N (%) or median (IQR)
***Participant***	
Total	833
Female	296 (36)
Age at screening (in years)	30 (26, 36)
Monthly income (USD)	39 (12, 114)
Education (in years)	8 (6, 12)
Married to study partner (yes, no)	791 (95)
Number of children with study partner	0 (0–2)
Couple aware of discordance status for > 1 month (yes, no)	675 (82)
Duration of discordance awareness (in months)	1 (1, 3)
Disclosed discordance status to anyone (yes, no)	291 (35)
Probable depression^a^ [21]	85 (10)
Desires children now or in future (yes, no)	619 (74)
Problematic alcohol consumption^b^ [22]	169 (20)
Relationship satisfaction score^c^ [23]	26 (24, 28)
Believes PrEP makes sex safe from HIV (yes, no/maybe/not-applicable)	358 (43)
Has fears or concerns about taking daily PrEP (yes, no)	88 (11)
Preference for HIV prevention	
Prefer partner start ARVs	167 (20)
Prefer to use daily PrEP	665 (80)
Had some perceived risk of getting HIV from partner (yes, no)	492 (59)
Frequency of sex in the partnership with < 100% condom use in past month	2 (0, 5)
***Partner living with HIV***	
Probable depression^a^	140 (17)
Problematic alcohol consumption^b^	152 (18)
Believes PrEP makes sex safe from HIV (yes, no/maybe/not applicable)	393 (47)

^a^Scoring an average of ≥1.75 on the Hopkins Depression Symptoms checklist was considered probable depression

^b^A participant who provides a positive response to any one of the four Rapid Alcohol Problems Screen (RAPS4) questions was considered to have problematic alcohol consumption

^c^The relationship satisfaction score ranges from 0 to 40 with higher values indicating more satisfaction

### Completion of the PrEP as a bridge to ART strategy

Of the participants whose partner started ART, just over half (*n* = 436, 52%) completed the 6-month PrEP as a Bridge to ART strategy. A total of 397 participants discontinued early because of a sustained ≥ 28-day interruption in electronic adherence data. However, of these, 79 (59%) resumed PrEP after a median of 50 days (IQR 35, 72) following the interruption.

### Predictors for completion of the PrEP as a bridge to ART strategy

As shown in Table 2, after adjusting for potential confounders, older age was associated with increased odds of completing the PrEP as a Bridge to ART strategy (aOR 1.1 [95% CI: 1.0, 1.3; *p* = 0.008] per 5 year increments), whereas each additional child with the study partner was associated with a 10% decrease in the likelihood of completing the strategy (aOR: 0.9 [95%CI: 0.8, 1.0]; *p* = 0.019).

**Table 2:** Correlates of completing the 6-month “PrEP as a bridge to ART” strategy among couples in which the partner living with HIV initiated ART (*n* = 833)

		Univariable		Multivariable	
Characteristics	N (%) or Median (IQR)	95% CI	*p*-value	95% CI	p-value
Completed bridge to ART	436 (52)				
***Participant***					
Female	142 (33)	0.8 (0.6, 1.1)	0.06	1.0 (0.7, 1.4)	0.90
Age at screening (per 5 years)	30 (26, 38)	1.1 (1.0, 1.2)	0.007	1.1 (1.0, 1.3)	0.008
Education (years)	8 (6, 12)	1.0 (0.9, 1.0)	0.18	1.0 (0.9, 1.0)	0.31
Married to study partner	417 (96)	1.4 (0.7, 2.5)	0.35		
Number of children with study partner (per additional child)	0 (0,1)	0.9 (0.9, 1.0)	0.087	0.9 (0.8, 1.0)	0.019
Couple had been aware of discordance status for > 1 month	347 (80)	0.8 (0.6, 1.1)	0.18	0.9 (0.6,1.3)	0.43
Probable depression^a^ [15]	35 (8)	0.6 (0.4, 1.0)	0.031	0.8 (0.5, 1.3)	0.42
Desires children now or in future	325 (75)	1.0 (0.7, 1.4)	0.92		
Problematic alcohol consumption^b^ [16]	78 (18)	0.7 (0.5, 1.0)	0.072	0.8 (0.5, 1.1)	0.2
Relationship satisfaction score^c^ [17]	26 (24, 28)	1.0 (1.0, 1.1)	0.62		
Learning that the partner has HIV while you do not was hard	–	0.8 (0.7, 0,9)	0.002	1.0 (0.8, 1.1)	0.52
Discovery of discordance created challenges	–	0.8 (0.7, 0.9)	< 0.001	0.9 (0.7, 1.1)	0.28
Managing serodiscordance well now	–	1.3 (1.1, 1.5)	0.011	1.1 (0.9, 1.4)	0.27
PrEP makes sex safe from HIV	188 (43)	1.0 (0.8, 1.3)	0.93		
No fears nor concerns about daily PrEP	34 (8)	1.9 (1.2, 2.9)	0.007	1.5 (1.0, 2.4)	0.070
Preference for HIV prevention					
Prefer partner start ARVs	76 (17)	Ref		Ref	
Prefer to use daily PrEP	360 (83)	1.4 (1.0, 2.0)	0.047	1.3 (0.9, 1.8)	0.15
Had some perceived risk of getting HIV from partner	257 (59)	1.0 (0.8, 1.3)	0.91		
High risk sexual behavior^d^ (sex + < 100% condom use)	2 (0, 5)	1.0 (1.0, 1.1)	0.058		
***Partner living with HIV***					
Probable depression^a^ [15]	65 (15)	0.8 (0.5, 1.1)	0.13	0.8 (0.5, 1.2)	0.23
Problematic alcohol consumption^b^ [16]	66 (15)	0.7 (0.5, 0.9)	0.015	0.8 (0.6, 1.2)	0.34
PrEP makes sex safe from HIV	204 (47)	1.0 (0.7, 1.3)	0.81		

^a^ Scoring an average of ≥ 1.75 on the Hopkins Depression Symptoms checklist was considered probable depression

^b^A participant who provides a positive response to any one of the four Rapid Alcohol Problems Screen (RAPS4) questions is considered to have problematic alcohol consumption

^c^The relationship satisfaction score ranges from 0 to 40 with higher values indicating more satisfaction

^d^ The number of sexual acts without a condom during the past month

### Continuation of PrEP beyond recommendations to stop

Of the 436 participants who completed the 6-months of PrEP as a Bridge to ART strategy, 191 (44%) continued PrEP thereafter according to strategy recommendations (i.e., because of concerns about the partner’s ART adherence, immediate fertility intentions, or reporting other partners with unknown HIV/ART status). One hundred seventy (39%) stopped PrEP as recommended, 60 (14%) continued to use PrEP for a median of 5 months (IQR 3, 7) beyond the initial 6 months and 15 (3%) had missing data. The reasons for continuing PrEP use were as follows: 23 (38%) indicated a desire for ongoing HIV prevention (e.g., citing discomfort or inability to negotiate condom use with the primary or other partners), 14 (23%) had not taken PrEP since last scheduled visit, 12 (20%) cited other reasons, and 5 (8%) wanted to support their partner living with ART; data were missing for the remaining 6 (10%).

### Predictors of continuation of PrEP beyond the PrEP as a bridge to ART strategy

As indicated in Table 3, after adjusting for potential confounders, more education and preference for taking PrEP (as opposed to the partner living with HIV taking ART as the means for preventing infection) were associated with continuing PrEP beyond 6 months (aOR 1.1 [95%CI: 1.0, 1.2]; *p* = 0.029 and aOR 3.6 [95%CI: 1.2, 11.2]; *p* = 0.026, respectively), whereas participants who reported to be managing their serodiscordant relationship well and those who believed that PrEP made sex safe from HIV were less likely to extend PrEP use beyond the recommendation (OR 0.6 [95%CI: 0.4, 1.0]; *p* = 0.046 and aOR 0.5 [95%CI: 0.2, 0.9]; p = 0.026, respectively).

**Table 3:** Correlates of PrEP use beyond the recommendation to stop (*n* = 230)

Characteristics		Univariable		Multivariable	
	N (%) or Median (IQR)	OR (95% CI)	p-value	OR (95% CI)	p-value
Stopped as recommended	60				
***Participant***					
Female	10 (17)	0.4 (0.2, 0.9)	0.034	0.6 (0.2, 1.5)	0.26
Age at screening (per 5 years)	29 (25, 35)	0.8 (0.7, 1.0)	0.015	0.8 (0.7, 1.0)	0.076
Education (years)	8.5 (8, 12)	1.1 (1.0, 1.2)	0.001	1.1 (1.0, 1.2)	0.029
Married to study partner	58 (97)	1.6 (0.3, 7.8)	0.55		
Number of children with study partner	0 (0,1)	0.8 (0.7, 1.1)	0.12	1.2 (0.9, 1.6)	0.22
At enrolment couple had been aware of discordance status for > 1 month	45 (75)	1.2 (0.6, 2.3)	0.68		
Probable depression^a^ [15]	5 (8)	0.9 (0.3, 2.7)	0.91		
Desire children now or in future	51 (85)	2.9 (1.3, 6.2)	0.008	2.2 (0.8, 6.3)	0.15
Problematic alcohol consumption^b^ [16]	14 (23)	1.4 (0.7, 2.9)	0.34		
Relationship satisfaction score^c^ [17]	27 (23, 28)	1.0 (0.9, 1.1)	0.54		
How difficult was it for you to learn that your partner has HIV while you do not	–	1.2 (0.9, 1.6)	0.18	0.9 (0.6, 1.4)	0.66
How much of a challenge did this discovery create for your relationship?	–	1.3 (0.9.1.8)	0.12	1.2 (0.8, 1.8)	0.50
How well are you managing the situation now?	–	0.6 (0.4, 0.9)	0.025	0.6 (0.4, 1.0)	0.046
PrEP makes sex safe from HIV	22 (37)	0.6 (0.3, 1.0)	0.055	0.5 (0.2, 0.9)	0.026
No fears or concerns about daily PrEP	54 (90)	1.1 (0.4, 3.0)	0.80		
Preference for HIV prevention					
Prefer partner start ARVs	6 (10)	ref		ref	
Prefer to use daily PrEP	54 (90)	2.0 (0.8, 5.3)	0.12	3.6 (1.2, 11.2)	0.026
Had some perceived risk of getting or transferring HIV to partner	39 (39)	1.2 (0.7, 2.2)	0.55		
High risk sexual behavior ^d^ (sex + < 100% condom use)	4 (2, 7)	1.0 (1.0, 1.1)	0.40		
***Partner living with HIV***					
Probable depression^a^ [15]	9 (15)	1.1 (0.5, 2.5)	0.87		
Problematic alcohol consumption^b^ [16]	4 (7)	0.3 (0.1, 1.0)	0.040	0.6 (0.2, 1.9)	0.36
PrEP makes sex safe from HIV	28 (47)	0.9 (0.5, 1.6)	0.72		

^a^ Scoring an average of ≥ 1.75 on the Hopkins Depression Symptoms checklist was considered probable depression

^b^A participant who provides a positive response to any one of the four Rapid Alcohol Problems Screen (RAPS4) questions is considered to have problematic alcohol consumption

^c^The relationship satisfaction score ranges from 0 to 40 with higher values indicating more satisfaction

^d^ The number of sexual acts without a condom during the past month

## Discussion

In this prospective, open-label study of integrated ART and PrEP use for HIV prevention among mutually disclosed HIV serodiscordant couples in Kenya and Uganda, just over half (52%) of the HIV-uninfected partners completed the PrEP as a Bridge to ART strategy. That is, PrEP was used for at least 6 months after the partner living with HIV initiated ART and was virally suppressed. Of those who were encouraged to stop PrEP at the end of the bridge period, 74% stopped. Given that only four participants acquired HIV and the study estimated a reduction in HIV transmission by 95% [21], this PrEP delivery strategy could be considered a success. However, counseling about this strategy during implementation should take into account the individual participant characteristics that were associated with more or less PrEP use than expected.

That older participants were more likely to complete the PrEP as a Bridge to ART strategy is not surprising. Indeed, several studies have found higher adherence to PrEP among older participants [5, 16, 17, 22]. Of note, “older” in this study predominantly means individuals in their 30s and 40s. Increased adherence with increased age likely reflects normal neurocognitive development in that younger people tend to be biased toward the present rather than focused on the prevention of an infection that may or may not happen in the future. Rather, younger people typically direct more thought and effort toward day-to-day concerns, such as food or emotional and physical satisfaction [23–25]. As individuals age, they are better able to focus on future goals and thus take medications on a regular basis and adhere to clinical recommendations. Tailoring PrEP delivery to the needs of younger people in their daily lives may improve uptake and adherence to PrEP [26]. Older age may also be an indicator for stable partnership, although it is not clear if stability in relationships influences adherence to clinical recommendations.

More complex is the finding that having more children with the study partner was associated with a lower likelihood of completing the PrEP as a Bridge to ART strategy. With ART, higher parity has been showed to be associated with an increased probability of imperfect adherence following delivery [27]. However, to our knowledge, such a relationship has not been established with PrEP. While our finding could reflect decreasing fertility desire and/or sexual activity within the partnership, neither of these factors were correlated with the number of children in the partnership or associated with completion of the bridge to ART strategy. Alternatively, the number of children could simply reflect alternative priorities beyond PrEP or HIV prevention in general. Further qualitative work will be important in understanding this association.

It is also not surprising that individuals who were managing their serodiscordant relationship well and those who believed that PrEP made sex safe were more likely to stop PrEP when counseled to do so. Participants who were managing their serodiscordant relationship well may have supported their partner in taking ART and/or had a high degree of trust in the relationship, correlating with high confidence in their partner’s ability to take ART well and achieve viral suppression. As Ware et al. [19] noted, support from partners reinforces success in adherence and viral suppression. Those who believed that PrEP made sex safe likely trusted the clinical advice they had received about taking PrEP and may have equally trusted the recommendation to stop it.

Regarding continued PrEP use, participants who were more educated were more likely to continue PrEP use beyond the recommendation to stop. Higher education levels have previously been shown to be associated with autonomy in decision-making among patients [28]. Other research, however, has found no correlation between education and adherence to instructions [29]. Continued PrEP use among participants who preferred to take PrEP (as opposed to their partner taking ART as the means to prevent HIV transmission) is logical and may reflect uncertainty regarding the U=U messaging [30]. Indeed, 23 of the 60 who continued PrEP use cited ongoing HIV prevention as their reason for continuing. These participants likely also had few concerns about PrEP itself or the effort involved in taking it. Interestingly, most participants preferred PrEP over ART, yet relatively few believed PrEP makes sex safe (90 and 37%, respectively). This finding may indicate a strong desire for individual control of their own HIV risk [31] even if complete confidence in effectiveness is lacking. Given that over one-quarter of participants continued PrEP beyond the PrEP as a Bridge to ART strategy, open discussions about individual desires and goals for PrEP use should be included in programmatic settings.

The low incidence of seroconversions despite many participants not completing the PrEP as a Bridge to ART strategy suggests a high degree of prevention-effective adherence during the study, as has been previously reported [5]. In other words, participants took PrEP long enough and well enough to cover their risk of HIV exposure. Potential explanations for the shorter-than-anticipated need for PrEP include rapid viral suppression in their partner taking ART or dynamic changes in risk (e.g., use of another HIV prevention tool such as condoms or lack of sex with the partner living with HIV). The alignment of PrEP use during periods of risk is especially plausible for the participants who did not complete the 6-month bridge owing to 28+ day interruptions, most of whom later resumed PrEP use. A high degree of prevention-effective adherence is also supported by the 26% of participants who continued PrEP beyond the recommendation to stop. These participants likely continued PrEP use because of some perceived on-going risk for HIV acquisition that was not otherwise captured. Elsewhere, research conducted in the United States and Australia also found higher adherence among participants reporting more risky behaviors [32–34]. These findings bode well for the use of the concept of prevention-effective adherence in the global rollout of PrEP.

That said, the concept of prevention-effective adherence was not fully developed at the time of the study. Rather, the risks and need for PrEP were explained to participants through the PrEP as a Bridge to ART strategy. The ability to navigate personalized use of PrEP will need to be explored in future work in which counseling is purposefully guided by prevention-effective adherence, as is happening in an ongoing study of PrEP use among young women in Kenya (NCT01140633). Importantly, qualitative work from this study identified some concern about the safety of the PrEP as a Bridge to ART strategy when viral load monitoring is not available [30, 35]. Participants who stopped PrEP still expressed fear for risk of infection and were likely to use other methods of prevention such as increased condom use, prioritization of fidelity in relationship, altering of sexual practice to minimize risk of infection, and establishing confidence in their partners ART adherence. Additional research is on-going regarding the self-identification of need for PrEP [36]. Promotion of the U=U campaign in this setting will be instrumental in disambiguating the fact that persons who are living with HIV and adherent to medication are therefore likely virally suppressed and will not infect their partner [7]. Care providers should also encourage serodiscordant couples to share information, such as adherence behavior and viral load results, which may inform the HIV-negative partners of the necessity or lack thereof for PrEP.

This study has a number of strengths, including a large sample size from two countries, objective electronic adherence measurements, and rich information on socio-behavioral factors relevant to PrEP use. It also has limitations. First, our outcome definitions may not be precise. The electronic adherence data was used to define PrEP use yet pill bottle openings do not necessarily reflect pill ingestion and misclassification may occur (e.g., due to device non-use). Additionally, all self-reported data, including our outcome of continuing PrEP beyond recommendations to stop which for some participants was self-reported, may be subject to social desirability bias. Importantly, we could only assess the demographic and socio-behavioral factors collected in the study. We do not have information about the decision-making process itself (e.g., desire for autonomy or trust in the clinical recommendations) that may affect completion of a clinical or public health strategy and may be challenging within couples [37]. These factors may be influential and could vary by culture, population, and/or setting. Indeed, our study findings can only be generalized to mutually disclosed serodiscordant couples with access to ART and PrEP free-of-charge. As with all observational studies, there is the possibility of residual confounding from unmeasured variables. Lastly, ART adherence by the partner living with HIV, which was not accounted for, may have influenced PrEP use and risk for HIV transmission.

## Conclusion

This analysis indicates that PrEP use guided by the PrEP as a Bridge to ART strategy was sufficient to achieve a high degree of HIV prevention. Future studies should assess this strategy in routine clinical settings with clear messaging about prevention-effective adherence and U=U, particularly among young PrEP users. The influence of more readily available viral load testing (e.g., with point-of-care technology [38] will also be important to explore. Counseling about this strategy should consider the factors that were associated with more or less PrEP use than expected.

## Data Availability

The datasets used and or analyzed during the current study are available on reasonable request. Data requests may be sent to icrc@uw.edu.

## Abbreviations

aOR: Adjusted odds ratio
ART: Antiretroviral therapy
HIV: Human Immunodeficiency virus
PrEP: Pre-exposure prophylaxis
